# Low T3 syndrome as a prognostic factor in patients in the intensive care unit: an observational cohort study

**DOI:** 10.5935/0103-507X.20220024-en

**Published:** 2022

**Authors:** Carla Daniele Nascimento Pontes, Juliane Lúcia Gomes da Rocha, Janaina Maria Rodrigues Medeiros, Bruno Fernando Barros dos Santos, Paulo Henrique Monteiro da Silva, Janine Maria Rodrigues Medeiros, Gabriela Góes Costa, Isabella Mesquita Sfair Silva, Daniel Libonati Gomes, Flávia Marques Santos, Rosana Maria Feio Libonati

**Affiliations:** 1 Núcleo de Medicina Tropical, Universidade Federal do Pará - Belém (PA), Brazil.; 2 Instituto de Ciências da Saúde, Universidade Federal do Pará - Belém (PA), Brazil.; 3 Instituto de Letras e Comunicação, Universidade Federal do Pará - Belém (PA), Brazil.

**Keywords:** Euthyroid sick syndrome, Mortality, Prognosis, Critical care, Triiodothyronine, Triiodothyronine, reverse, Intensive care units

## Abstract

**Objective:**

To assess euthyroid sick syndrome as a prognostic factor in patients in the intensive care unit; to detect factors that may affect mortality; and to develop an equation to calculate death probability.

**Methods:**

This was a longitudinal, observational, nonconcurrent cohort study developed in the intensive care unit of *Fundação Santa Casa de Misericórdia do Pará*. One hundred adults with no prior documented endocrinopathy were submitted to a 20mL blood sample collection for the measurement of thyroid stimulating hormone, free tetraiodothyronine, free triiodothyronine and reverse triiodothyronine.

**Results:**

Most patients were female, aged 20 to 29 years. Most patients who died were older (median age of 48 years), and euthyroid sick syndrome was present in 97.5% of them. Euthyroid sick syndrome was related to death, comorbidities, age and length of stay in the intensive care unit (median of 7.5 days).

There was an association between lower thyroid stimulating hormone and death. Patients with free triiodothyronine levels below 2.9pg/mL were more likely to die; reverse triiodothyronine rates were above 0.2ng/mL in those who died. Free triiodothyronine had greater sensitivity and accuracy, and reverse triiodothyronine had greater specificity to predict mortality. Based on the results and cutoff points, a multiple logistic regression formula was developed to calculate the probability of death.

**Conclusion:**

The main limitation of this study is the fact that it was conducted in a reference hospital for maternal and child care; therefore, there was a greater number of female patients and, consequently, a sampling bias existed. However, opportune measurement of free and reverse triiodothyronine levels in critical patients and application of the proposed equation are suggested.

## INTRODUCTION

Thyroid dysfunction related to a decrease in triiodothyronine (T3) synthesis is closely linked to poor prognosis in severe systemic diseases.^(1-3)^ These findings are related to neuroendocrine dysregulation in critically ill patients in whom there is a reduction in T3 and tetraiodothyronine (T4) synthesis without stimulation of thyroid stimulating hormone (TSH) secretion.^(4)^ This is due to metabolism in critically ill patients, which generates suppression of thyrotrophin releasing hormone (TRH) gene expression and, consequently, underregulation of pituitary-hypothalamic-thyroid axis activity.^(5)^

The changes observed in these situations have been classified as “euthyroid sick syndrome” (ESS), which can occur in up to 70% of hospitalized patients with severe systemic disease, including patients with severe liver, lung, and kidney disease, postoperative status, physical trauma, acute infections, psychiatric disorders, and malnutrition.^(6)^ This syndrome consists of low serum levels of total T3 and/or free T3 (FT3), high reverse T3 (rT3) levels, and normal or low TSH, total T4 and free T4 (FT4) levels.^(7,8)^ The most clinically important and most common finding is a T3 decrease, which justifies the other term “low T3 syndrome”, but variants of this condition that present with deficient T4 are also related to worse prognosis.^(9)^

In this regard, the relationship between decreased T3 levels and poor prognosis is consistent and can be used as an indicator for the severity of systemic diseases.^(3,10)^ Thus, this study aimed to evaluate low T3 syndrome as a prognostic factor in patients hospitalized in the intensive care unit (ICU), to identify variables that may interfere with death probability and to develop a flowchart to calculate the chances of this outcome.

## METHODS

This is a longitudinal, observational, nonconcurrent cohort study conducted at *Fundação Santa Casa de Misericórdia do Pará* (FSCMPA). The research project was approved by the Research Ethics Committee of FSCMPA, opinion 2,568,906, in March 2018.

Between August and December 2018, a total of 248 patients were hospitalized in the ICU of FSCMPA, of which 100 patients, aged 18 years or older, who consented to participate in the research or had their legally responsible representative provided consent were included in the study. Patients diagnosed with documented endocrinopathy were excluded. However, patients who used medications that interfered with thyroid function, such as amiodarone, lithium, dopamine, androgens and steroids, were not excluded.

**Figure 1:**
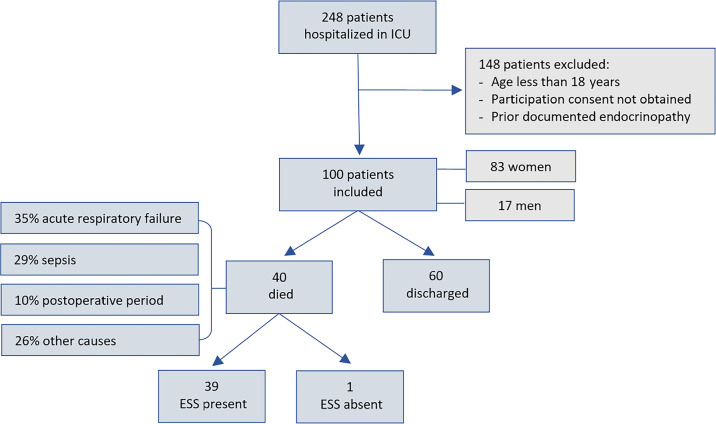
Overview of patients admitted to the intensive care unit who were included in the study. ICU - intensive care unit; ESS - euthyroid sick syndrome.

Considering an average monthly turnover of 50 patients in the ICU (approximately 250 patients in 5 months), a confidence interval of 95%, and a prevalence of ESS of 70%, according to the literature,^(6)^ a sample size calculation of 139 patients was obtained. The fact that it was not possible to reach the minimum sample for the study is a limitation, but the other patients unfortunately did not meet the inclusion criteria, that is, patients aged 18 years or older with no prior documented endocrinopathy, who were admitted to the ICU during the study period and agreed to participate by providing written informed consent (ICF) (Figure 1).

Although the study had been conducted in a maternal and child reference hospital, it is important to point out that FSCMPA adult ICU covers the treatment of male patients as well, whose main causes of admission include sepsis, respiratory failure, liver failure, cholangitis, postoperative care, shock, heart disease, and neoplasia.

The case group consisted of individuals exposed to ESS, while the control group was composed of individuals not exposed to ESS. Data were obtained by collecting 20mL blood samples, all of which were collected at 5 a.m. for the measurement of TSH, FT4, FT3 and rT3 hormones. Thirty-one percent of the patients had their samples collected within the first 12 hours after admission to the ICU, 26% between 12 and 24 hours, 19% between 24 and 48 hours, 10% between 48 and 72 hours, and 14% above 72 hours after ICU admission.

For the analysis of TSH, FT4 and FT3, blood centrifugation was performed within 3 hours after collection, and the samples were stored at a temperature of 68 to 77°F. The samples were sent on the same day for laboratory analysis, during which the hormones were dosed using the chemiluminescence technique in SIEMENS® kits. For rT3 analysis, the material was stored at a temperature of 68 to 77°F and analyzed within a maximum of 24 hours by radioimmunoassay using a ZenTech® kit. The reference values for analysis were as follows: TSH, 0.36 to 5.40µUI/mL; FT4, 0.74 to 1.72ng/d; FT3, 2.9 to 4.2pg/mL; and rT3, 0.09 - 0.2ng/mL. ESS was defined as FT3 below the reference value and rT3 above the reference value, regardless of TSH and FT4.

The descriptive variables collected were sex, causes of referral to the ICU, comorbidities, presence of ESS, outcome and causes of hospitalization leading to death. In descriptive statistics, measures of central tendency and dispersion were analyzed. The G-test and chi-square test were used to assess qualitative variables, and the Mann-Whitney test was used to assess quantitative variables. The cutoff point was calculated, which determined the sensitivity, specificity and accuracy of variables that showed a significant association with death outcome in those patients diagnosed with ESS. Variables showing significant correlation were used in multiple logistic regression to analyze death outcome , which was the dependence variable. Descriptive and analytical statistics were performed using BioEstat® 5.3 software,^(11)^ with adoption of significance level α = 0.05 or 5% for decision-making.

For the flowchart elaboration, guiding the investigation of ESS, cutoff points with greatest significance and accuracy were identified to obtain more accurate prognostic prediction. Initially, patients admitted to the ICU within at least 24 hours were screened for FT3 due to its greater sensitivity and accuracy in diagnosing the syndrome. If FT3 was decreased, the investigation continued with assessment of rT3 levels.

If FT3 was decreased and rT3 was elevated, diagnosis of ESS was given, and the patient’s mortality assessment continued with analysis of other variables that were most related to death; i.e., age, length of stay in ICU and presence of comorbidities.

From these data, the five variables were classified according to their cutoff points. For variables considered risk factors (rT3, age, length of stay in ICU and presence of comorbidities), 0 was adopted when the variable was below the cutoff point, and 1 was assigned when the variable was above the cutoff point. When FT3 was considered a protective factor, 1 was adopted when the variable was below the cutoff point, and 0 was assigned when the variable was above the cutoff point. From the cutoff points, a logistic regression formula was first calculated to identify the value of logit Pi. Then, this value was used in the formula for the death probability. The final value was expressed in decimal form and was multiplied by 100 to identify the death probability expressed in percentage values.

As an adjustment for potential confounders, such as age and comorbidity, multiple logistic regression was performed. Although the variable comorbidity did not show a significant association with death, it was decided to keep it in the death probability equation, since the association is significant in univariate analysis.

## RESULTS

From August to December 2018, a total of 248 patients were hospitalized in the ICU of FSCMPA, of which 100 patients who consented to participate in the research or had their legally responsible representative provided consent were included in the survey; the majority of participants were female (83%), which was statistically significant. The most prevalent age group was 20 to 29 years (Chisquare test, p < 0.0001), and the mean age was 34 years. Regarding causes of referral to the ICU, the main cause was eclampsia (30%), followed by sepsis (26%), respiratory failure (15%), postoperative period (12%), shock (7%), heart disease (4%), neoplasia (3%) and others (3%). Of all hospitalized patients, 59% had comorbidities associated with the initial cause of hospitalization (Chi-square test, p > 0.05), including neoplasia, acquired immunodeficiency syndrome, pneumopathy, chronic kidney disease, hemorrhage, cirrhosis, and congestive heart failure.

Most patients (36%) remained hospitalized in the ICU during the period of 1 to 6 days. The average length of hospitalization was 8 days. For the diagnosis of ESS, there was a prevalence of the syndrome in 72% of patients (Chi-square test, p < 0.0001).

Most patients had normal TSH levels (83%), normal FT4 levels (82%), normal FT3 levels (75%) and increased rT3 levels (74%); the last two were statistically relevant (Chisquare test, p < 0.05).

Regarding outcomes, 60 patients (60%) were discharged from the ICU, and 40 (40%) died (Chi-square test, p < 0.0001). With death as an outcome, of the patients who died, 39 (97.5%) had euthyroid syndrome. Among the causes of hospitalization leading to death, acute respiratory failure (35%), sepsis (29%) and the postoperative period (10%) were the most prevalent. Furthermore, patients who died were older, with a median age of 48 years, compared to a median of 25 years among those who did not die, showing an association between death and age (Mann-Whitney test, p < 0.0001). There was also an association between death and length of stay in the ICU, with a median time of 7.5 days, compared to a median of 4 days among those who did not die (Mann-Whitney test, p < 0.0001).

The prevalence of ESS was higher in males (94%), while for females, the prevalence was 67.5% (Fisher’s exact test, p = 0.035). Mean age was higher for patients who had ESS, as well as for male patients. Only one male patient, aged 61 years, did not have ESS (Table 1). ESS was identified in 90% of people with comorbidities and in 61.1% of those without comorbidities (Fisher’s exact test, p = 0.0001). Stratified analysis between comorbidities and death with the presence or absence of ESS demonstrated that when ESS was absent, there was no correlation between comorbidities and death (Fisher’s exact test, p = 0.85). In contrast, when ESS was present, there was a highly significant association between comorbidities and death (Fisher’s exact test, p = 0.00000035).

**Table 1:** Patient profiles according to the presence or absence of euthyroid sick syndrome

		Euthyroid sick syndrome	
	ESS Group	Control Group	p value
Mean age - male	42.38 ±1.54	-	< 0.0001^*^
Median age - male	45.5 ±1.2	-	< 0.0001^*^
Mean age - female	35.66 ± 19.86	25.55 ± 5.93	< 0.0001 and 0.096^*^
Median age - female	21 ± 19.86	25 ± 5.93	< 0.0001^*^
Mean TSH (µUI/mL)	1.79 ± 1.6	2.57 ± 2.2	0.0250†
Mean Free T4 (ng/dL)	1.05 ± 0.3	1.03 ± 0.3	0.8659†
Mean Free T3 (pg/mL))	1.66 ± 0.5	2.41 ± 0.6	< 0.0001†
Mean Reverse T3 (ng/mL)	0.70 ± 0.3	0.22 ± 0.1	< 0.0001†
Presence of comorbidities	73	27	0,0001‡

EES - euthyroid sick syndrome; TSH - thyroid stimulating hormone; T4 - tetraiodothyronine; T3 - triiodothyronine.

* Chi-square test; †Mann-Whitney Test; ‡Fisher Test.

The association between TSH levels and the presence of ESS was significant, demonstrating that lower TSH values are directly related to the diagnosis of ESS. The correlation between FT4 levels and the presence of ESS was statistically insignificant (Table 1). The conditional probability (ROC curve) of TSH, FT4, FT3 and rT3 was also calculated according to death outcome. The assessment of TSH levels and its correlation with progression to death showed that TSH levels were lower in patients who died, with a median of 1.09µUI/mL, while the median of those who did not die was 1.74µUI/mL (Mann-Whitney test, p < 0.023). The assessment of the conditional probability between TSH and death revealed a cutoff point of 1.32uUI/mL for TSH, with a sensitivity, specificity and accuracy of 62.5%, 63.3% and 62.98%, respectively. In contrast, there was no association between FT4 and death, and the conditional probability between FT4 and death revealed a cutoff point of 0.97ng/mL, with a sensitivity, specificity and accuracy of 52.5%, 61.7% and 58.02%, respectively, revealing that the test was nonspecific and correlated with mortality.

**Figure 2:**
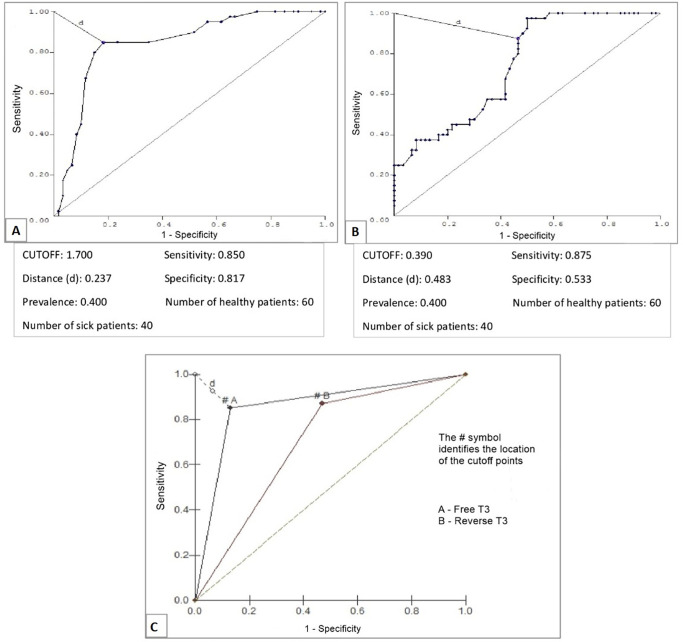
ROC curves for free triiodothyronine (A), reverse triiodothyronine (B) and both (C) as tests for predicting death in patients admitted to the intensive care unit. T3 - triiodothyronine.

The analysis between FT3 and death showed that patients who died had lower FT3 levels, with a median of 1.5pg/mL for those who died and 2pg/mL for those who survived (Mann-Whitney test, p < 0.0001), and the assessment of conditional probability between FT3 and death revealed a cutoff point for FT3 of 1.7pg/mL (Figure 2A). The correlation between rT3 and death showed that there was a higher value of this hormone in patients who died, with a median of 0.56ng/mL for those who died and 0.35ng/mL for those who survived (Mann-Whitney test, p = 0.0001); the conditional probability between rT3 and death revealed a cutoff point of 0.97ng/mL (Figure 2B).

When comparing TSH, FT4 and FT3, it was noted that FT3 had better performance, revealing it as the best predictor of death. Among TSH, FT4 and rT3, rT3 performed better than the other hormones in correlating with death. However, when comparing FT3 and rT3 as predictors of death, it was noted that FT3 was superior (Figure 2C).

The evaluation of ESS as a risk factor for death showed a death prevalence of 54.17% among those who had ESS and 3.57% among those who did not, with an odds ratio of 31.9 (p < 0.0001). Thus, the chance of adult patients hospitalized in the ICU progressing to death is 31.9 times greater among patients with ESS. The relative increase in risk was 50.60%, showing that for every two adult patients admitted to the ICU with ESS, one will progress to death. Analyzing only patients who had been diagnosed with ESS and the outcome of death, FT3 had the greatest sensitivity and accuracy, and rT3 was the most specific hormone.

This shows that if rT3 is normal, the chance of survival of the patient is greater (Table 2).

**Table 2:** Relation between thyroid hormones and death

Death	p value	Sensitivity	Specificity	Accuracy	PPV	NPV
TSH	0.0543	56.4	70.6	63	68.7	58.5
Free T4	0.30	51.3	64.7	57.54	62.48	53.69
Free T3	< 0.0001^*^	82.10	79.4	80.84	79.47	79.47
Reverse T3	0.36	38.5	85.3	60.31	75.01	54.76

PPV - positive predictive value; NPV - negative predictive value; TSH - thyroid stimulating hormone; T4 - tetraiodothyronine; T3 - triiodothyronine.

* Mann-Whitney test. Values expressed as %.

**Figure 3:**
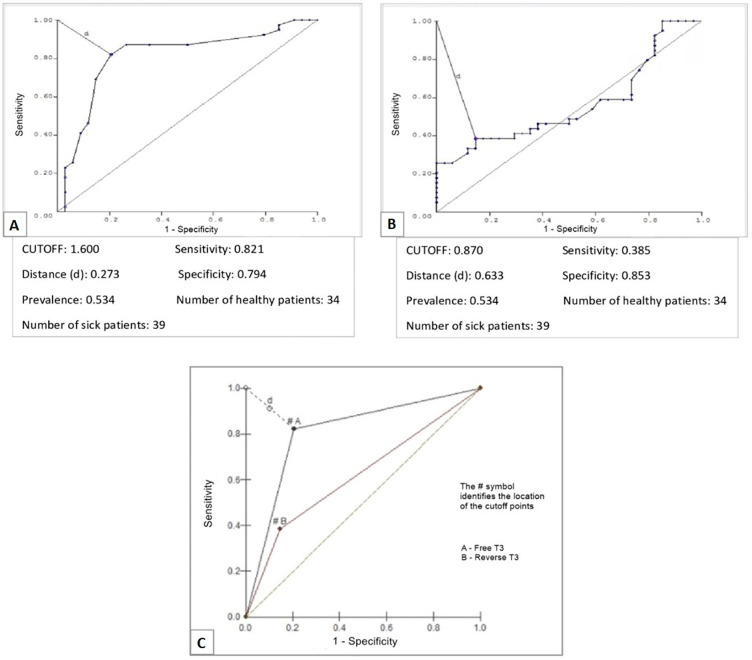
ROC curves for free triiodothyronine (A), reverse triiodothyronine (B) and both (C) and their relations with death in the presence of euthyroid sick syndrome in patients admitted to the intensive care unit. T3 - triiodothyronine.

The conditional probability between FT3 and death revealed a cutoff point of 1.6pg/mL for FT3 (Figure 3A; Table 2). The evaluation of conditional probability between rT3 and death revealed a cutoff point of 0.87ng/mL (Figure 3B; Table 2). Finally, when comparing FT3 and rT3 as predictors of death, FT3 was superior (Figure 3C). As FT4 and TSH did not present significant differences between patients with ESS who progressed to death, the conditional probability of these hormones was not calculated.

The correlation between length of stay in the ICU and death in patients with ESS was significant. A median of 8 days was observed in patients with ESS who died, which was in contrast to a median of 4 days for those who did not die (Mann-Whitney test, p < 0.0001). For the conditional probability, the cutoff point was 7 days, with a sensitivity, specificity and accuracy of 61.5%, 82.4% and 71.24%, respectively.

When comparing the ages of patients with ESS who died, a positive correlation was found between death and age. A median of 48 years was observed among patients with ESS who died, in contrast to a median of 23 years for those who did not die (Mann-Whitney test, p < 0.0001). For the conditional probability, the cutoff point was 39 years, with a sensitivity, specificity and accuracy of 61.5%, 94.1% and 76.7%, respectively.

Considering variables significantly associated with death, multiple logistic regression was performed; FT3, rT3, age and length of stay in the ICU were considered based on their cutoff points. Death was significantly associated with the variables age, FT3 and length of stay. The association with FT3 was inversely proportional, showing that lower values are related to higher mortality (Table 3). From these data, the regression formula was calculated: [Logit Pi = -3.1176 + (2.8899 x 0) + (2.6958 x 1) + (0.6120 x 0) + (2.9073 x 0) + (1.9419 x 0)].

**Table 3:** Odds of death according to the analyzed variables

		Multiple logistic regression		
Death X variables	β coefficient	p value	Odds ratio	95%CI
Length of stay	2.88	0.0047	17.99	2.43 - 133.59
Age	2.69	0.0186	14.81	1.57 - 139.92
Comorbidities	0.61	0.4639	1.84	0.36 - 9.48
Free T3	2.90	0.0023	18.30	2.82 - 118.99
Reverse T3	1.94	0.0562	6.97	0.95 - 51.17

95%CI - 95% confidence interval; T3 - triiodothyronine.

**Figure 4:**
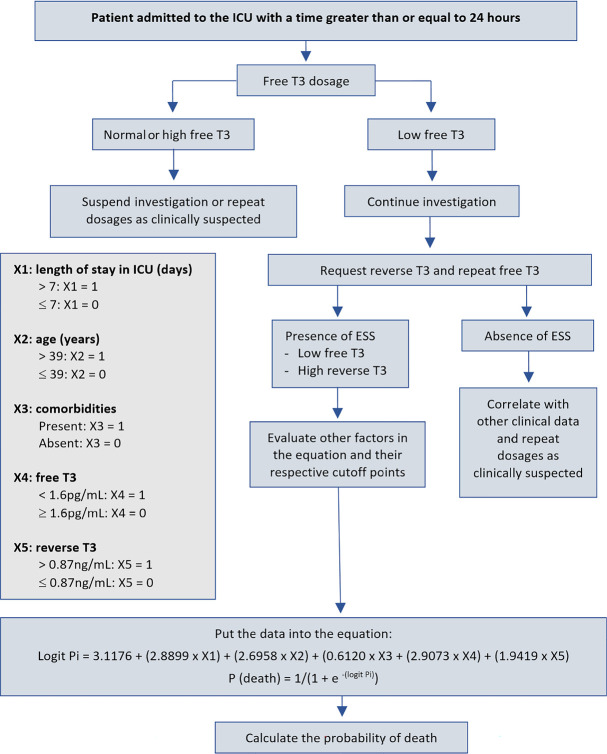
Suggested flowchart to estimate prognosis in critically ill adult patients admitted to the intensive care unit with the diagnosis of euthyroid sick syndrome. ICU - intensive care unit; T3 - triiodothyronine; ESS - euthyroid sick syndrome.

Therefore, the flowchart illustrated in figure 4 is suggested to serve as a calculation of prognosis in critically ill adult patients hospitalized in the ICU with a diagnosis of ESS. Based on this flowchart (Figure 4), an application for mobile phones called the “Low T3 Death Probability Calculator (LowT3DPC)” was developed to help health professionals calculate death probability in patients admitted to the ICU with low serum levels of FT3 and high levels of rT3, characterizing euthyroid sick syndrome (https://github.com/danlibs/LowT3DeathProbabilityCalculator).

## DISCUSSION

### Epidemiological profile

During the study period, most patients admitted to the ICU were females. This diverges from evidence reported in the literature, which emphasizes that patients admitted to the ICU are mostly males. Commonly, the hospitalization of men is associated with trauma, surgery, cardiovascular complications and infections, which testifies to the higher occupancy rate by this group in the ICU.^(12)^

Regarding age, the most prevalent age group overall was younger than reported in the literature, in which the average age of patients admitted to the ICU was generally between 55 and 65 years or older. This includes patients with smaller available reserves, more comorbidities and a greater chance of worsening their clinical condition. However, although most of the study sample was composed of young people, older ages were directly related to death outcomes.^(13)^

In this context, older ages were observed among patients who had ESS compared to those who did not have the syndrome. Literary evidence that confirms this information indicates that older patient age is associated with a greater chance of being affected by acute injuries and a higher mortality rate and shows a more direct association with disease severity, as well as being related to impaired functional status prior to admission to the ICU.^(14)^

These findings regarding the most prevalent gender and age may occur because FSCMPA is a reference hospital for maternal and child care. As a result, more female patients are admitted, especially those of childbearing age and with high-risk pregnancy; therefore, a greater potential exists for serious conditions that require referral to the ICU. Despite the prevalence of female participants, ESS was associated with male sex; however, there is no evidence in the literature that shows a defined epidemiological profile regarding gender, age or ethnicity of patients at higher risk of developing ESS and, as a consequence, a higher mortality rate.

### Causes of referral and length of stay

The main reasons for referring patients to the ICU were eclampsia, sepsis and respiratory failure. These findings are similar to other studies of obstetric populations that define hypertensive and hemorrhagic causes as major reasons for hospitalization, followed by sepsis, trauma and respiratory causes.^(15)^ There was also an association between comorbidities and ESS. In cases already diagnosed with ESS, comorbidities were associated with risk of death.

Regarding the length of stay in the ICU, more hospitalization days confer a greater risk to critically ill patients, either due to disease progression or a greater risk of pathologies related to health care. The association between length of stay and death showed the greatest risk starting from the seventh day of hospitalization. Literature is divergent as to the exact number of days that add greater morbidity and mortality, ranging from 3 to 7 days in most studies, but all are emphatic at showing a direct relationship between longer stay and higher mortality.^(16-18)^

### Mortality associated with euthyroid sick syndrome

The overwhelming majority of patients who progressed to death had ESS diagnosis, with respiratory failure, sepsis and a complicated postoperative period being the main causes of death. A previous study demonstrated that the FT3 level is directly related to surfactant production, alveolar fluid clearance and alveolar surface tension regulation.^(19)^ Furthermore, it is known that septic patients have a greater chance of thyroid disorders such as ESS, which is associated with higher morbidity and mortality.^(20)^ Thus, ESS may help to perpetuate metabolic disturbances and may even be directly related to the main causes of death in the hospital environment, specifically the ICU.

Conditional probability analysis of thyroid hormones with death revealed that lower TSH, lower FT3 and higher rT3 levels were more related to death, while FT4 was not. Although prognosis depends on the underlying disease, the comparison of hormonal dosages showed that FT3 levels are more accurate for the prognosis of critically ill patients. A study conducted at a university hospital in China involving 188 leukemic patients demonstrated that low FT3 is related to an increased inflammatory state and worse leukemia prognosis.^(21)^

### Limitations and future perspectives

Despite showing an association, the observational method does not allow for determining a causal relationship between ESS and mortality in critical patients. In other words, it is still not possible to define whether thyroid disorder is the cause or if mortality is associated with a set of alterations caused by another main disorder. However, previous studies have shown the importance of ESS in several pathologies, such as acute myocardial infarction, major burns, cancer, cirrhosis and chronic kidney disease.^(22-24)^

In that regard, the use of the tool presented in this study can be interesting for the prognostic assessment of critical patients. However, we recognize the need for its improvement, serving as a basis for further research.

In addition, it is important to emphasize that supplementation with thyroid hormones is not recommended under any circumstance.

Despite the minimum sample size of 139 people, it was only possible to obtain consent from 100 participants. Furthermore, the fact that the research was conducted in a reference hospital in maternal and child care ensured that a greater number of female patients were admitted, and the main cause of ICU admission was childbirth-related conditions such as eclampsia; thus, sampling bias may have occurred. *Fundação Santa Casa de Misericórdia do Pará* is also a reference in high-risk pregnancies and thus associated with the highest mortality in young women. This can generate results of low similarity when talking about external validation, considering that patients admitted to the ICU are mostly males.^(12)^ In addition, it is important to consider that the applicability of the proposed tool can be impaired, considering the limited availability of thyroid hormone measurements in some hospitals. The absence of prognostic indices in the study analysis, such as the Simplified Acute Physiology Score 3 (SAPS 3) and Acute Physiology and Chronic Health Evaluation (APACHE), can also be described as a limitation, despite not being the objective of this study.

## CONCLUSION

Euthyroid sick syndrome was present in 72% of the population, and 40% progressed to death. When euthyroid sick syndrome was present, there was a highly significant association between comorbidities and death. Higher reverse triiodothyronine, lower thyroid stimulating hormone and free triiodothyronine levels were found in patients who died, with the latter being the best test for predicting death. The chance of progressing to death was greater among patients with euthyroid sick syndrome.

However, the assessment of euthyroid sick syndrome is often neglected in the vast majority of intensive care units, despite the importance of this syndrome having already been reported in previous studies showing free triiodothyronine as an independent marker for mortality in critical patients, and the relationship between euthyroid sick syndrome and worst outcome in different scenarios. Therefore, when these tests are available, opportune measurement of free and reverse triiodothyronine levels in critically ill patients hospitalized in the intensive care units is suggested, as well as the application of the proposed equation to help assess their prognosis.
